# Yarning about fall prevention: community consultation to discuss falls and appropriate approaches to fall prevention with older Aboriginal and Torres Strait Islander people

**DOI:** 10.1186/s12889-017-4628-6

**Published:** 2017-08-01

**Authors:** Caroline Lukaszyk, Julieann Coombes, Norma Jean Turner, Elizabeth Hillmann, Lisa Keay, Anne Tiedemann, Cathie Sherrington, Rebecca Ivers

**Affiliations:** 1 The George Institute for Global Health, PO Box M201, Missenden Road, Sydney, NSW 2050 Australia; 2 Sydney Medical School, University of Sydney, Sydney, NSW 2006 Australia; 3 Illawarra Shoalhaven Local Health District, Suite 2 Level 2, 67-71 King Street, Warrawong, NSW 2502 Australia; 4 Mingaletta Aboriginal and Torres Strait Islander Corporation, 6 Sydney Avenue, Umina, NSW 2257 Australia; 5 School of Nursing and Midwifery, Flinders University, Sturt Rd, Bedford Park, SA 5042 Australia

**Keywords:** Indigenous, Aboriginal and Torres Strait Islander, Fall prevention, Ageing, Yarning circles

## Abstract

**Background:**

Fall related injury is an emerging issue for older Indigenous people worldwide, yet few targeted fall prevention programs are currently available for Indigenous populations. In order to inform the development of a new Aboriginal-specific fall prevention program in Australia, we conducted community consultation with older Aboriginal people to identify perceptions and beliefs about falls, and to identify desired program elements.

**Methods:**

Yarning Circles were held with Aboriginal and Torres Strait Islander people aged 45 years and over. Each Yarning Circle was facilitated by an Aboriginal researcher who incorporated six indicative questions into each discussion. Questions explored the impact of falls on Yarning Circle participants, their current use of fall prevention services and investigated Yarning Circle participant’s preferences regarding the design and mode of delivery of a fall prevention program.

**Results:**

A total of 76 older Aboriginal people participated in ten Yarning Circles across six sites in the state of New South Wales. Participants associated falls with physical disability, a loss of emotional well-being and loss of connection to family and community. Many participants did not use existing fall prevention services due to a lack of availability in their area, having no referral provided by their GP and/or being unaware of fall prevention programs in general. Program elements identified as important by participants were that it be Aboriginal-specific, group-based, and on-going, with the flexibility to be tailored to specific communities, with free transport provided to and from the program.

**Conclusions:**

Older Aboriginal people reported falls to be a priority health issue, with a significant impact on their health and well-being. Few older Aboriginal people accessed prevention programs, suggesting there is an important need for targeted Aboriginal-specific programs. A number of important program elements were identified which if incorporated into prevention programs, may help to address the rising burden of falls.

## Background

Community consultation is important in the planning and implementation of community-based health programs [1]. It allows for communication between community members, program developers and funding bodies, ensuring community-based health programs address the issues affecting the health and wellbeing of local populations. Community consultation has shown to be particularly valuable in the development of health programs for Indigenous populations worldwide [2]. Consultation not only allows for programs to respond to the unique needs and priorities of Indigenous populations, but importantly, allow Indigenous people to become active partners in identifying key problems and solutions for themselves and their communities [3], facilitating self-determination.

Falls and fall-related injury are becoming a growing concern for global Indigenous populations as they age [4–6]. For Australia’s Aboriginal and Torres Strait Islander population, fall injury rates have increased by an average of 10.2% per year from 2007 to 08 to 2010–11, compared to a 4.3% average annual increase for other older Australians [7]. Falls are now the second most common cause of injury for all Aboriginal and Torres Strait Islander people in Australia [8], with the highest fall-injury rates reported for females aged 65 years and above, and males aged 60–64 years [9]. For older people, there is a high likelihood that a fall can cause injury, potentially resulting in significant functional decline or even permanent disability [10]. Experiences of past falls can also lead to an increased fear of falling, preventing people from performing daily tasks and limiting their independence [11].

Despite high and rapidly increasing fall-injury rates, there is little knowledge about the impact of falls in Aboriginal and Torres Strait Islander people in Australia, or in older Indigenous people worldwide [12]. Further, while there are a variety of fall prevention programs currently run in community settings, it is uncertain whether these programs are accessed by older Indigenous people or whether they are effective or acceptable for these populations. Previous research has shown that successful health programs implemented in Indigenous communities have different content, structure and methods of delivery than those developed for the general population [13]. Indigenous leadership and community ownership of health programs ensures they answer to local community needs, can be modified readily to suit changing community priorities and are run corresponding to local belief systems and practices [14, 15].

Yarning Circles are a method of storytelling, education and preserving cultural knowledge, used for thousands of years by Indigenous people in Australia, Canada and North America [16]. ‘Research Topic Yarning’ is well-documented and has been previously used to gain community input for the design/delivery of community-based health programs for Indigenous populations [17, 18]. It is compared to a semi-structured interview and described as ‘a yarn with a purpose’ [19]. It enables researchers to learn from the stories and experiences of Yarning Circle participants in relation to a specific issue or question.

Within this study, Yarning Circles were used by our team of Indigenous and non-Indigenous researchers to explore three key areas: 1) investigate the impact of falls on the health and wellbeing of older Aboriginal and Torres Strait Islander people; 2) assess the level of existing knowledge older Aboriginal and Torres Strait Islander people have on fall prevention; and 3) to identify desirable elements of a fall prevention program from the perspective of older Aboriginal and Torres Strait Islander people.

As the majority of the New South Wales (NSW) Aboriginal and Torres Strait Islander population is Aboriginal (97.2%), this population will be referred to as ‘Aboriginal’ in this manuscript.

## Methods

### Participants

Invitations to host Yarning Circles were extended to a selection of Aboriginal health and community services across NSW, frequently accessed by older Aboriginal people. These services were identified through consultation with the project’s Aboriginal Steering Committee. The study was promoted by posters displayed at each service and through active recruitment by service staff. People interested in participating either registered with service staff or contacted the project’s Aboriginal research officer (JC). A greater burden of chronic health conditions affects Aboriginal and Torres Strait Islander people at younger ages when compared to the general population [20]. Due to this, Aboriginal and/or Torres Strait Islander people aged 45 years and above were eligible to register for the study, rather than the cut-off age of 65 years typically used to classify older adults.

### Data collection

Each Yarning Circle was held at a venue and time convenient to participants and host service staff. An Aboriginal facilitator trained in qualitative research methods guided each Yarning Circle (JC), ensuring cultural safety was maintained during each discussion. A research officer was present to take notes and make observations (CL). There was time reserved before the Yarning Circles for the visiting project team to meet with Yarning Circle participants over morning tea, build rapport and establish a welcoming environment. All Yarning Circle discussions began with both the participants and the project team introducing themselves and sharing their family origins. The facilitator introduced six open-ended questions into each Yarning Circle over time (Table 1) and prompted Yarning Circle participants to continue the discussion when appropriate.

**Table 1:** Question guide for Yarning Circles

1.	Has anyone had a fall recently? Can you talk about what happened?
2.	Is it important to find ways to prevent falling?
3.	Are you aware of any fall prevention programs in your community?
4.	Would you attend an Aboriginal-specific fall prevention program if it was available?
5.	Are there any things that would/do stop you from attending a fall prevention program? Do you have any ideas about how these things could be addressed?
6.	*[Laminated cards with pictures representing existing fall prevention programs distributed]* Are there any parts of these programs that you would like to see incorporated into a falls program for Aboriginal people?

### Data analysis

**Fig. 1:**
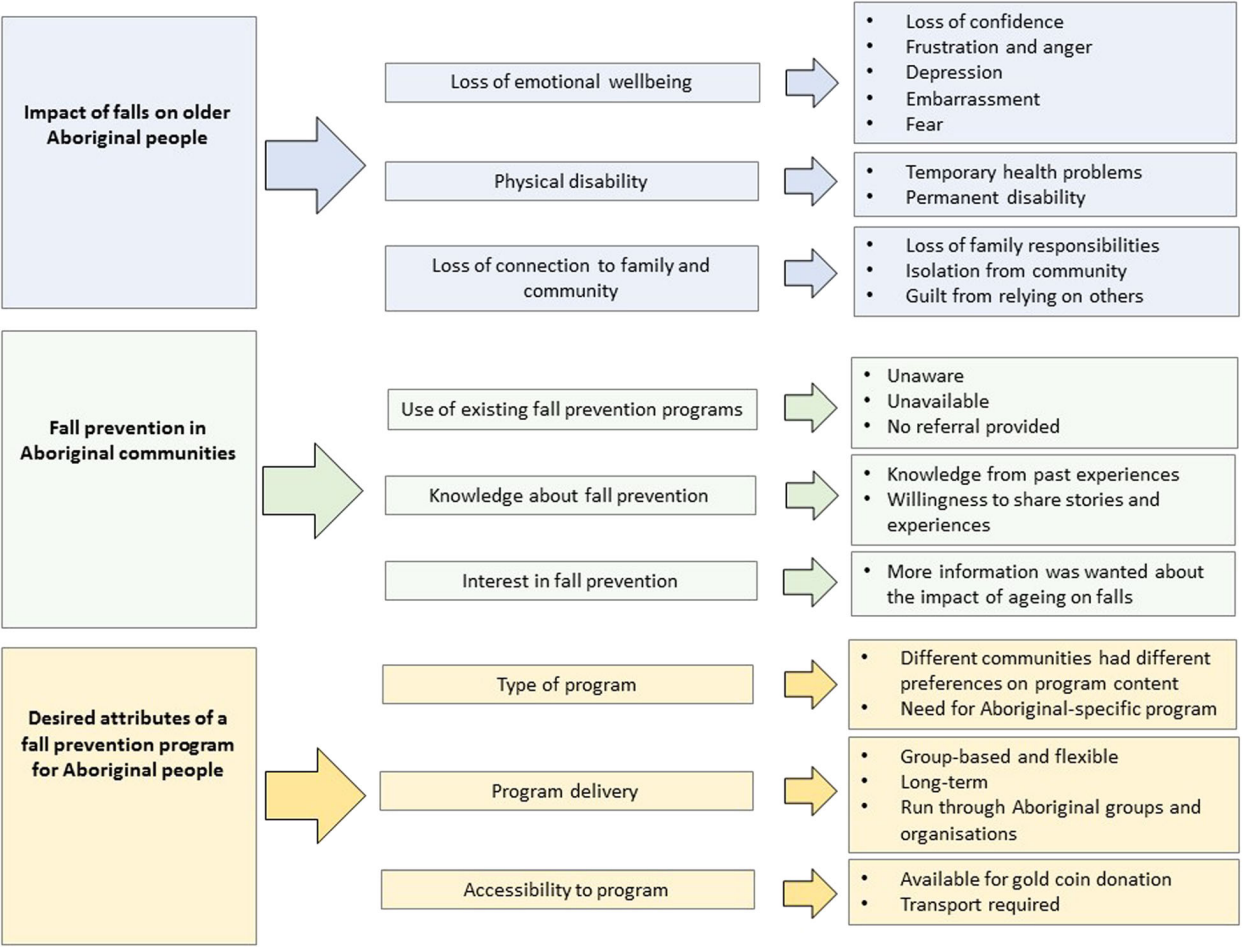
Themes which emerged from Yarning Circle transcripts surrounding falls and fall prevention for older Aboriginal people, grouped into broader categories. A summary of the impact of falls on older Aboriginal people, the current status of fall prevention within Aboriginal communities and suggestions for the design and delivery of an appropriate fall prevention program for Aboriginal populations, from the perspectives of older Aboriginal people

Each Yarning Circle ran for between 30 min and 1 h, was audio recorded and transcribed verbatim. Data were managed in NVivo10 (QSR International PTY Ltd) software. Conventional content analysis was used to analyse the transcripts due to the variety of topics addressed in the study which could not be confined to fit within a pre-existing methodological framework [21]. By avoiding the use of preconceived categories for coding data, the core messages that emerged from the Yarning Circles were able to be captured and presented as key findings. Each Yarning Circle transcript was independently and sequentially coded by three researchers, two of whom are Aboriginal (CL, JC, EH). Each researcher repeatedly read all transcripts, immersing themselves in the data. Following coding, discussion and comparison took place, where themes were grouped under broader categories presented in Fig. 1. A senior Aboriginal community spokesperson who was present during multiple Yarning Circles (NJT) reviewed the results, provided feedback and was closely involved with the writing of this manuscript. This manuscript is reported in line with the COREQ (Consolidated criteria for reporting qualitative research) statement, supporting transparency in reporting qualitative research [22].

### Ethical approval

The study received ethical approval from the Aboriginal Health & Medical Research Council of NSW (AH&MRC) (1084/15). Written consent was given by all study participants for Yarning Circles to be audio recorded and transcribed, with de-identified results permitted to be published.

## Results

A total of 10 Yarning Circles were held with 76 participants (16 males, 60 females) across six sites in Sydney, the Central Coast, Central West and Illawarra Shoalhaven, NSW (Table 2). Each Yarning Circle consisted of 7 to 10 participants. When over 10 participants were present at a site, multiple Yarning Circles were held.

**Table 2:** Yarning Circle host organisation type, location and number of Yarning Circle participants

Host service type	Location	Number of male participants	Number of female participants
Aboriginal chronic care group	Central Coast	1	9
Aboriginal Medical Service	Central Coast	3	5
Aboriginal community organisation	Central Coast	3	15
Aboriginal chronic care group	Illawarra Shoalhaven	8	14
Aboriginal Land Council	Central West	1	6
Aboriginal community organisation	Sydney	0	11
Total		16	60

### Impact of falls on older aboriginal people

#### Physical disability

Many Yarning Circle participants shared stories of falling, often with multiple falls reported by one person. Temporary health problems and permanent disabilities that had occurred due to past falls were discussed. One female participant spoke of mobility loss caused by a fall-related leg injury, leading to weight gain and a further loss of balance, recalling that ‘one thing seemed to lead to another’.

“[After my fall] I couldn’t walk the way I wanted to walk to lose the weight. And because I’ve put the weight on, it’s impacted on my health.” *(F).*

Rehabilitation services required post fall-related injury were reported to be inflexible, too expensive and difficult to access.

#### Loss of emotional wellbeing

Yarning Circle participants spoke of feeling angry, embarrassed and frustrated after having a fall. They shared stories of losing mobility and/or losing confidence, preventing them from independently performing activities of daily living or being active in the community. This had the potential to lead to isolation and depression.

“It’s pretty frustrating and it makes you angry, makes you sad, you get depressed ‘cause you can’t do what you used to do.” *(M).*

Some Yarning Circle participants described feeling guilty for relying on family participants to care for them after a fall.

“I felt so useless because even when I did eventually come home [following a fall-related hospitalisation] I couldn’t do a lot. It was a horrible feeling because the kids had to come over and bring meals, my brother brought a few meals, and it’s a horrible feeling, horrible.” *(F).*

#### Loss of connection to family and community

The majority of Yarning Circle participants spoke of acting as carers for their extended families. This was seen as an extremely valued and important responsibility. Those who had experienced health problems due to a fall spoke of feeling uncomfortable in becoming dependant on their children or siblings for care. Stories were shared of family members overcompensating in supervising a loved one following a fall, further limiting their independence.

“No, I do miss walking. But I’m not to be trusted out alone anymore. So I have keepers.” *(F).*

A fear of falling was reported to make Yarning Circle participants less likely to try new things. This prevented people from attending social occasions, leading to social isolation and loneliness.

“I think falls shake your confidence. So regardless of whether it’s a minor fall, a major fall or whatever, it can affect your mental attitude to things. So, if it rocks your confidence you’re less likely to try and do other things.” *(M).*

### Fall prevention in Aboriginal communities

#### Use of fall prevention programs

Generally, Yarning Circle participants reported they were unaware of existing fall prevention programs. Few participants spoke vaguely about past involvement in short-term programs run through larger health services however, they were unsure as to why they were referred or how the program was meant to benefit them.

“I went to a falls program that one Wednesday, and all they did was make me stand up and sit down. I refused to do anything.” *(F).*

#### Knowledge about fall prevention

Within each Yarning Circle, participants shared a great deal of knowledge about fall prevention amongst themselves, despite very few having exposure to formal fall prevention education. ‘Lessons had been learnt’ by participants who had previously fallen and were willing to share their experiences with the group. They went on to explain the precautions they now took to prevent further falls.

“You become conscious of it too as far as other people are concerned.” I caught my wife the other day on the rocking chair dusting up [the ceiling]. I said to her, “What are you doing up there?” *(M).*

#### Interest in fall prevention

Yarning Circle participants were interested to understand how aged-related changes to their physical ability increased their risk of falling. Some participants spoke of reporting a fall to their GP, but no referral to a fall prevention program being given. Others spoke of long wait times to access regularly run, short-term programs.

“I’m not a faller at all and in the last six months I’ve had two really, really bad falls, and I’ve ended up in hospital on both occasions and I can’t believe that it was me. I’m starting to think that if I don’t start doing something now, it’s going to be worse for me when I do get to that stage where, yeah…” *(F).*

### Desired attributes of a fall prevention program for Aboriginal people

#### Type of program

Different communities voiced different preferences for program content, ranging from dance, Tai Chi to light gym work. There was a consensus that the program shouldn’t be physically demanding, allowing everyone to feel confident in participating.

It was important to Yarning Circle participants that specific issues relating to older Aboriginal people were acknowledged through the program. Participants wanted to show that Aboriginal communities were able take responsibility for their health, in their own way. Each community was enthusiastic to make the program ‘their own’ by tailoring it to their interests and health needs.

*“We could stand up and show what we've been doing, you know? Advertise it, let people see that we actually are trying to get healthy. That'd show them that we are doing something.” (M).*

Aboriginal programs were discussed as more inclusive of all people and as places of less judgement. They were seen as culturally safe and more flexible than programs developed for the general population. People felt relaxed among their own community, where everyone was seen as equal. Others saw Aboriginal programs as an opportunity to learn more about local Aboriginal history and culture, with the potential of meeting other members of their family who they may have not met before.

“I have found, just from my experience that the Aboriginal programs are a lot friendlier, a lot more relaxed, not so rigid, and not so judgmental. And you don’t need that judgmental stuff. You just need people who have the same or similar problems as you that are willing to accept you as you are, and you’re going to find some common ground with being able to help them, and they help you. And I think that’s one of the major differences.” *(F).*

#### Program delivery

Having the program delivered in a group setting was unanimously important to Yarning Circle participants. Participants wanted the group to be friendly, where they had an opportunity to share stories and feelings openly and safely. ‘Getting out of the house’ was associated with mental health benefits as it presented an opportunity to meet new people. Happiness and emotional wellbeing were seen as important outcomes of the program. Group-based programs were thought to motivate people to return on a regular basis and to introduce challenge via friendly competition between participants.

“I’d like a group because you can enjoy it and you can have a laugh and a joke or carry-on a bit. It’ll do more good that than sitting there trying to do it yourself at home. If it’s up to me to do something at home, I’d say bugger it.” *(F).*

Yarning Circle participants wanted the program to be flexible and self-paced, catering to the abilities and needs of the local community. Participants preferred that the program should have no age limit so that partners, children and carers were welcome to attend. It was preferred for the program to be held within existing community groups, ideally through local Aboriginal organisations.

There was a strong preference for an on-going long-term program rather than a short-term program. Yarning Circle participants shared concerns of missing a short-term program due to other family, health or community commitments. It was reported to take time to ‘get used to’ a new program, and to form friendships between program participants. It was also seen as important to follow-up with program participants over the long term.

“This six weeks or this eight weeks thing, it’s just no good for the Koori [Aboriginal] community because people get sick. People drop out through winter. People drop out for various reasons but they can come back, pick up where they left off and continue on. You can’t offer Koori communities short term fixes because it doesn’t fix anything.” *(F).*

#### Accessibility to program

The majority of Yarning Circle participants were willing to pay a small fee from AUD$1 to AUD$10 to participate in a fall prevention program. It was important for participants to see value in the program; participants wanted to know how the program could benefit them and wanted it delivered by professionally trained staff. Other financial commitments, particularly those surrounding family, were often seen as a higher priority. Despite this, paying a weekly fee to prevent falls was seen as valuable.

*“Well when you think about it in the long run: you’re paying $5 a week for a group as opposed to not being as strong and having falls. When you have a fall you lose so much independence in the way of washing, drying, all that sort of stuff. Five dollars a week in the scheme of things is not a huge amount.” (F).*

Yarning Circle participants were concerned that issues with transport could leave people who are unable to reach the program independently becoming further isolated. Some people reported to not be physically able or confident to catch public transport, while others had little to no public transport available in their area.

*“We’ve got a lot of older people that want to do these exercise classes but just can’t get to the place whether it be ‘cause they don’t have a licence or do have a licence but don’t have a car, or can’t afford busses.” (F).*

Yarning Circle participants from existing community groups were able to assist each other with transport to a service or a program, highlighting the importance of community cohesion.

## Discussion

The Yarning Circles highlighted concerns around falls and the significant impact falls have on social and community life for older Aboriginal people. The importance of community consultation was demonstrated, with many issues discussed surrounding falls and fall prevention being unique to the older Aboriginal population. In line with previous research [2, 3], a strong and consistent theme that emerged from the Yarning Circles was the need for fall prevention services specifically designed and delivered for Aboriginal people. Yarning Circle participants voiced a strong preference for a group-based program, tailored to suit local interests and health priorities. It was essential that all community members were included and able to participate in the program. The provision of transport as part of the program was considered important and a small donation was viewed as appropriate for program use.

The limited research available investigating falls and fall-related injury in older Indigenous populations suggests different patterns and outcomes of falls when compared to equivalent mainstream populations [23–26]. Despite this, many issues surrounding falls documented from general populations mirror those discussed by Aboriginal Yarning Circle participants. Common issues included sustaining serious injuries that cause chronic pain and disability, a loss of independence, loss of confidence, depression and developing a fear of falling [27–29]. The loss of family and community responsibilities were additional issues discussed by Yarning Circle participants.

The previous uptake of fall prevention programs by other older populations has been reported to be low. Typically, 10–50% of an eligible population participates in fall prevention interventions at a community level [29]. These low rates are associated with people not viewing themselves as ‘at risk’ of falling, or being unaware that falls are preventable. In previous studies, falls have been associated with a loss of control and seen as an indication of a transition into old age [30]. Previous studies have also documented older people as being very reluctant to discuss falls due to embarrassment. On the contrary, Yarning Circle participants were very willing to discuss personal stories of past falls within each group, listening to each other’s experiences with interest and providing suggestions on how to prevent future falls from occurring, or how to manage resulting health issues. A lack of awareness about fall prevention interventions emerged as the predominant reason for Yarning Circle participants not using existing fall prevention programs. Service providers working in Aboriginal aged care within NSW have provided similar feedback, stating that few older Aboriginal clients access fall prevention services as they are unaware they are available [31].

There was unanimous agreement by participants that the program should be Aboriginal-specific, acknowledging issues of particular importance and relevance to older Aboriginal people. Previous studies have identified a number of health and social issues that affect Indigenous populations and mainstream populations differently [32, 33], leading to different areas needing to be prioritised by health services and health programs for Indigenous communities [34]. Yarning Circle participants additionally expressed the need for a program with the flexibility to be customised to suit the diverse range of Aboriginal communities across the state, while remaining evidence-based and effective. Ensuring cultural safety through providing an Aboriginal-specific program was also stated as important by Yarning Circle participants. Previous studies have documented Indigenous people’s experiences of discrimination, judgement and communication difficulties when accessing mainstream health services [35–37].

The inclusive, group-based setting requested for a fall prevention program by Yarning Circle participants has previously been identified as an effective approach towards community participation and promoting community ownership of a program [38]. Community ownership has been reported as a key contributor to the success of Indigenous health services and health programs worldwide [39, 40]. Previous successful Indigenous-specific programs delivered in ‘safe and supportive group environments’ have led to a greater sense of participant well-being and support amongst group members [41]. The request for a long-term, ongoing program is not unique to this study. Many initiatives run in Aboriginal communities are a product of short-term grants which do not get funded in the long term [42]. Similar issues have been reported for the funding of Indigenous health services worldwide [39].

Costs associated with accessing health services have been reported as a barrier to their use by approximately one third of Australia’s Aboriginal population [43], particularly when costs are ongoing [44]. Nearly all existing fall prevention programs run in NSW charge an attendance fee ranging from a donation to $22 per session [45]. Although the majority of Yarning Circle participants agreed that a small donation was appropriate for program use, there were concerns that other community members may not be able to afford this and would therefore be excluded. Transport to and from regular program sessions introduces a secondary barrier to program use, particularly in remote communities. Long distance, poor roads and a lack of public transport cause people living remotely (particularly older people) to have a strong reliance on private and community transport options, which can be expensive and in high demand [46]. Transport availability and cost were highlighted by participants as a concern and ongoing programs would need to address this.

The strengths of this study include the involvement of Aboriginal people in all aspects of study design, participant recruitment, data collection, data analysis and manuscript preparation. To our knowledge, this is the first qualitative study which documents the views of older Indigenous people regarding healthy ageing, worldwide. A greater proportion of women participated in the study than men. This may mean that perspectives from older Aboriginal men may not have been appropriately considered. Furthermore, this study only reflects the views of Aboriginal community members from NSW, Australia. Although the results of this study are anticipated to be generalizable, repetition of this study within different Indigenous populations, both within Australia and internationally, would be valuable for comparison. Nonetheless, there are many similarities in the health and social issues that affect Indigenous populations worldwide and it is anticipated the findings of this study may inform the development of prevention programs for other older Indigenous populations.

## Conclusions

Yarning circles with older Aboriginal people facilitated important discussions of the impact of falls. Many Yarning Circle participants shared stories of falls impacting their health, well-being and connection to family and community. Existing mainstream fall prevention programs were generally not used by Yarning Circle participants due to their lack of availability in certain areas, no referral provided for fall prevention services by GPs and/or being unaware of existing programs. Despite few Yarning Circle participants receiving formal fall prevention education, significant knowledge was shared from past experiences and individual ideas for minimising fall risk and managing recovery from fall-related injury. Feedback from participants highlighted that an ongoing, Aboriginal-specific, group-based fall prevention program was preferred, that could be run through established Aboriginal organisations with the flexibility to be tailored to specific communities while remaining effective and evidence based. Multiple issues discussed by participants in relation to falls and fall prevention were unique to the older Aboriginal population and were not being met by existing services, highlighting the importance of community consultation, but also Aboriginal leadership and program ownership. The findings of this study will guide the development and implementation of appropriate fall prevention programs for older Indigenous populations worldwide.

## Abbreviations

AH&MRC: Aboriginal Health & Medical Research Council of NSW
COREQ: Consolidated criteria for reporting qualitative research
NSW: New South Wales
